# Severe cerebellitis as a manifestation of infliximab-associated overlap of neurosarcoidosis and myelin oligodendrocyte glycoprotein antibody-associated disease – case report

**DOI:** 10.3389/fimmu.2026.1759831

**Published:** 2026-06-01

**Authors:** Gregor Hütter, Maximilian Winkelhausen, Lars Tönges, Ralf Gold, Simon Faissner

**Affiliations:** Department of Neurology, St. Josef-Hospital, Bochum, Germany

**Keywords:** autoimmune neurology, cerebellitis, infliximab, neurosarcoidosis, TNF-α- inhibitor, MOGAD

## Abstract

**Background:**

Tumor necrosis factor-α (TNF-α) inhibitors such as infliximab are widely used in autoimmune disorders. Paradoxical inflammatory reactions including systemic sarcoidosis and neurosarcoidosis are increasingly recognized but remain rare.

**Case presentation:**

We report a 56-year-old male with ulcerative colitis under infliximab therapy presenting with acute dysarthria and gait instability. Neurological examination revealed truncal ataxia. Brain and spinal MRI were normal. Cerebrospinal fluid analysis showed lymphocytic pleocytosis, reduced glucose, increased lactate, and elevated protein with marked blood-brain barrier disruption. Sarcoidosis-associated markers (neopterin, ACE, IL-2 receptor) were elevated. Pulmonary CT and lymph node biopsy confirmed systemic sarcoidosis. Moreover, MOG-antibodies were detected in both serum and CSF. After initial empirical anti-infective therapy, high-dose corticosteroids were initiated, resulting in full recovery. Infliximab therapy was discontinued.

**Conclusion:**

This case demonstrates infliximab-associated overlapping autoimmunity with sarcoidosis, probable neurosarcoidosis and MOGAD presenting with acute cerebellitis. Clinicians should consider neurosarcoidosis and MOGAD in patients on TNF-α-inhibitors presenting with neurological deficits, even in the absence of radiological findings. Early discontinuation of the triggering agent and timely corticosteroid therapy are essential for favorable outcomes.

## Introduction

Tumor necrosis factor-α (TNF-α) inhibitors, including infliximab, are established therapeutic options in autoimmune disorders such as ulcerative colitis, rheumatoid arthritis, and psoriasis (1). Despite their efficacy, paradoxical inflammatory reactions have been reported, including sarcoidosis-like granulomatous disease and demyelinating syndromes. Neurological manifestations, although rare, may include optic neuritis, myelitis, cranial neuropathies, and neurosarcoidosis.

Cerebellar inflammation as a presentation of infliximab-associated neurosarcoidosis has to the best of our knowledge not yet been reported. This case highlights the diagnostic complexity and therapeutic implications of acute cerebellitis in the context of TNF-α-inhibitor therapy.

## Case presentation

A 56-year-old Caucasian male with ulcerative colitis under stable infliximab therapy, initiated 13 months prior onset of neurological symptoms, presented to the emergency department with acute onset of dysarthria and gait instability with inability to walk within three months after symptom onset. He reported markedly reduced walking distance (300 m maximum without support) and increasing frequency of falls. Neurological examination revealed no paresis or sensory deficits but pronounced cerebellar signs, including impaired finger-to-nose and heel-to-shin coordination, severe truncal ataxia, and inability to maintain an upright seated position. Tandem gait was severely impaired.

Emergency cranial computed tomography (CT) was unremarkable. Laboratory testing showed no abnormalities. Lumbar puncture revealed lymphocytic pleocytosis (100 cells/µl), decreased glucose, elevated lactate, and marked protein elevation. Pending exclusion of infection, empirical antimicrobial therapy (ceftriaxone, ampicillin, aciclovir) was initiated. Comprehensive microbiological testing revealed no evidence of an infectious etiology. Cerebrospinal fluid (CSF) multiplex PCR was negative for common viral and bacterial meningoencephalitis pathogens, and additional single PCR assays, 16S-rDNA PCR including fungal pathogen, INH/RIF testing for M. tuberculosis, and Gram-stain for acid-fast bacilli were all unremarkable. Neurophysiological studies demonstrated prolonged latencies in tibial somatosensory evoked potentials (SSEP), bilateral visual evoked potentials (VEP) latency prolongation, and normal motor evoked potentials (MEP). Nerve conduction studies revealed axonal-sensory polyneuropathy. Optical coherence tomography (OCT) was unremarkable.

Follow-up cerebrospinal fluid (CSF) analysis revealed persistent pleocytosis (40/µl, predominantly lymphocytes). Immunological work-up detected a serum IgG/IgA gammopathy. Fluorescence-activated cell sorting (FACS) analysis showed >90% T-cells without malignant infiltration; cytology excluded malignancy. CSF demonstrated severe blood-brain barrier dysfunction without intrathecal antibody synthesis. Paraneoplastic antibody screening was negative. Anti-MOG antibodies were positive in serum (1:32) and confirmed in both serum and CSF by live-cell assay (serum 1:160; CSF 1:2), raising suspicion of MOG-associated disease (MOGAD).

However, sarcoidosis-associated markers in CSF (neopterin, ACE, IL-2 receptor) were also elevated. Magnetic resonance imaging (MRI) of brain and spinal cord was unremarkable. Whole-body CT revealed mediastinal and hilar lymphadenopathy, suspicious colonic wall thickening, and a hypodense liver lesion. Colonoscopy excluded neoplasia or active colitis. Liver MRI showed focal steatosis. Bronchoscopy with endobronchial ultrasound (EBUS) and bronchoalveolar lavage (BAL) revealed non-caseating granulomas in lymph node biopsies, confirming sarcoidosis. BAL CD4+/CD8+ ratio was 1.3.

High-dose intravenous corticosteroids followed by oral tapering led to rapid improvement, with complete resolution of dysarthria and ataxia. Infliximab was discontinued. At six-month follow-up under maintenance prednisolone (5 mg/day), neurological status was normal. CSF analysis showed no pleocytosis or protein elevation, while neopterin, soluble IL-2 receptor and ACE remained mildly elevated. Systemic inflammatory markers persisted at low levels, consistent with chronic systemic sarcoidosis without CNS involvement.

## Discussion

The presented case of acute cerebellitis during infliximab therapy for ulcerative colitis fits the spectrum of paradoxical sarcoid-like reactions under TNF-α blockade. Of note, the medication in the patient discussed here was initiated more than one year prior onset of neurological signs, in line with delayed complications of anti-TNF-α inhibitors having been described to occur more than five years after treatment initiation (2). Such reactions are considered a class effect and are hypothesized to arise from cytokine disequilibrium (e.g., interferon pathway skew) and altered T-cell regulation that favor granuloma formation (3). Clinically, pulmonary, cutaneous, and nodal disease predominate, but CNS involvement, including meningitis, cranial neuropathies, myelitis, and cerebellar syndromes, though less common, can be severe and diagnostically challenging (4). Of note, coincidental sarcoidosis cannot be fully included.

For diagnostic framing, the Neurosarcoidosis Consortium criteria stratify disease into possible, probable, and definite categories, integrating clinical, MRI, CSF, and histologic data; in patients without neural tissue biopsy, systemic non-caseating granulomas plus compatible neuroinflammatory findings support probable neurosarcoidosis (5). Our patient’s lymphocytic pleocytosis with hypoglycorrhachia and barrier dysfunction, elevated CSF neopterin/ACE/sIL-2R, systemic granulomatous histology, and complete steroid response align with this construct despite normal MRI; a scenario recognized in neurosarcoidosis and underscoring the need for multimodal work-up (4, 5).

The therapeutic paradox of infliximab in sarcoidosis is well documented: infliximab is effective for refractory neurosarcoidosis in observational cohorts and a recent meta-analysis, yet anti-TNF-α agents can also precipitate sarcoid-like disease in susceptible hosts (6). In pathology-confirmed neurosarcoidosis, infliximab is associated with clinical and radiologic improvement in most patients, but relapse and infection risks remain salient (6–8). Conversely, anti-TNF-α induced sarcoidosis typically improves upon drug withdrawal ± corticosteroids, supporting causality in paradoxical cases (3). Given the temporal relationship, systemic confirmation, biomarker profile, and full remission after high-dose steroids with infliximab cessation, infliximab-associated neurosarcoidosis, taking the positivity for MOG-antibodies into consideration, in part explains this presentation.

Cerebellar involvement in neurosarcoidosis is uncommon but reported, from mass-like lesions mimicking neoplasms to immune-mediated cerebellar ataxia, highlighting phenotype variability and the importance of excluding infection and neoplasm before immunotherapy (9, 10). Detection of MOG-IgG antibodies represented a relevant differential diagnosis, as TNF-α inhibitors are also linked to demyelinating events (11, 12). The 2023 diagnostic criteria for MOG antibody-associated disease (MOGAD) are formally fulfilled in this case (13); however, a sole attribution of the clinical presentation to MOGAD appears unlikely. The coexistence of MOG-IgG positivity with elevated CSF markers of sarcoidosis-associated inflammation and histologically confirmed non-caseating granulomas suggests a more complex immunopathogenesis. One plausible explanation is a “double-hit” mechanism in the context of anti-TNF-α therapy, as previously proposed (14). In this framework, MOG-IgG antibodies detected in both serum and CSF may represent an additional antibody-mediated immune response contributing to the neurological phenotype. However, the subsequent clinical course, characterized by complete remission, absence of relapses, and improvement of CSF findings, argues for a contribution of MOG-IgG-associated immune activity during the initial disease phase. This is supported by the literature since in a cohort of 138 patients with MOGAD 3.6% had no MRI lesion at the time of attack (15). Anti-TNF-α therapy may have triggered a broader dysregulation of immune homeostasis, leading both to granulomatous inflammation consistent with sarcoidosis-like disease and to the emergence of MOG-IgG autoantibodies. This interpretation is in line with the concept of paradoxical immune activation under TNF-α blockade, encompassing both granulomatous and demyelinating processes. Accordingly, the neurological presentation in this case may reflect an overlapping or combined mechanism, in which infliximab-induced sarcoidosis-like disease represents the primary driver, with a potential modulatory contribution from MOG-IgG-associated immune activity. Given that both neurosarcoidosis and MOGAD typically respond well to corticosteroid therapy, distinguishing their relative contributions in this context remains impossible. Overall, this case highlights the possibility that multiple immune-mediated mechanisms may coexist under TNF-α inhibition, complicating diagnostic attribution and underscoring the need for careful longitudinal assessment. Management principles include rapid infection exclusion, early high-dose corticosteroids, and removal of the offending anti-TNF-α agent when paradoxical sarcoidosis is suspected; steroid-sparing agents (e.g., methotrexate, azathioprine, mycophenolate) or B-cell depletion (rituximab) are options for refractory disease (4) with similar therapeutic approaches in MOGAD including also IvIG and tocilizumab.

In summary, this case emphasizes the importance of vigilance for neurological complications under TNF-α inhibitors. Cerebellitis, although rare, can represent a manifestation of drug-induced neurosarcoidosis and MOGAD. Early recognition, exclusion of infection, and timely immunotherapy are crucial to prevent permanent neurological deficits.

## Data Availability

The raw data supporting the conclusions of this article will be made available by the authors, without undue reservation.

## References

[1] Jang DI Lee AH Shin HY Song HR Park JH Kang TB . The role of tumor necrosis factor alpha (TNF-α) in autoimmune disease and current TNF-α inhibitors in therapeutics. Int J Mol Sci. (2021) 22(5):27192021. doi: 10.3390/ijms22052719. PMID: 33800290 PMC7962638

[2] Barbella G Antenucci P Rosa M Gozzi A Padroni M . Neurosarcoidosis-like reaction under TNF-α inhibitors: a case report and literature review of a paradoxical immune phenomenon. Neurological Sciences: Off J Ital Neurological Soc Ital Soc Clin Neurophysiol. (2026) 47(4):346. doi: 10.1007/s10072-026-08955-z. PMID: 41838193 PMC12992451

[3] Massara A Cavazzini L La Corte R Trotta F . Sarcoidosis appearing during anti-tumor necrosis factor alpha therapy: a new "class effect" paradoxical phenomenon. Two case reports and literature review. Semin Arthritis Rheum. (2010) 39:313–9. doi: 10.1016/j.semarthrit.2008.11.003. PMID: 19147181

[4] Bradshaw MJ Pawate S Koth LL Cho TA Gelfand JM . Neurosarcoidosis: pathophysiology, diagnosis, and treatment. Neurol Neuroimmunol Neuroinflamm. (2021) 8(6):e1084. doi: 10.1212/NXI.0000000000001084 34607912 PMC8495503

[5] Stern BJ Royal W Gelfand JM Clifford DB Tavee J Pawate S . Definition and consensus diagnostic criteria for neurosarcoidosis: from the Neurosarcoidosis Consortium Consensus Group. JAMA Neurol. (2018) 75:1546–53. doi: 10.1001/jamaneurol.2018.2295. PMID: 30167654

[6] Gelfand JM Bradshaw MJ Stern BJ Clifford DB Wang Y Cho TA . Infliximab for the treatment of CNS sarcoidosis: a multi-institutional series. Neurology. (2017) 89:2092–100. doi: 10.1212/wnl.0000000000005725. PMID: 29030454 PMC5711506

[7] Fritz D Timmermans WMC van Laar JAM van Hagen PM Siepman TAM van de Beek D . Infliximab treatment in pathology-confirmed neurosarcoidosis. Neurol Neuroimmunol Neuroinflamm. (2020) 7(5):e847. doi: 10.1212/nxi.0000000000000847. PMID: 32718952 PMC7413716

[8] Chaiyanarm S Satiraphan P Apiraksattaykul N Jitprapaikulsan J Owattanapanich W Rungjirajittranon T . Infliximab in neurosarcoidosis: a systematic review and meta-analysis. Ann Clin Transl Neurol. (2024) 11:466–76. doi: 10.1002/acn3.51968. PMID: 38087813 PMC10863903

[9] Kumar G Kang CA Giannini C . Neurosarcoidosis presenting as a cerebellar mass. J Gen Intern Med. (2007) 22:1373–6. doi: 10.1007/s11606-007-0272-7. PMID: 17619108 PMC2219770

[10] Mizushima K Yaguchi H Sato S Yabe I . Immune-mediated cerebellar ataxia with neurosarcoidosis. Intern Med. (2023) 62:2023–4. doi: 10.2169/internalmedicine.0784-22. PMID: 36384902 PMC10372285

[11] Kemanetzoglou E Andreadou E . CNS demyelination with TNF-α blockers. Curr Neurol Neurosci Rep. (2017) 17:36. doi: 10.1007/s11910-017-0742-1. PMID: 28337644 PMC5364240

[12] Bradshaw MJ Mobley BC Zwerner JP Sriram S . Autopsy-proven demyelination associated with infliximab treatment. Neurol Neuroimmunol Neuroinflamm. (2016) 3:e205. doi: 10.1212/nxi.0000000000000205. PMID: 26894207 PMC4747476

[13] Banwell B Bennett JL Marignier R Kim HJ Brilot F Flanagan EP . Diagnosis of myelin oligodendrocyte glycoprotein antibody-associated disease: International MOGAD Panel proposed criteria. Lancet Neurol. (2023) 22:268–82. doi: 10.1016/s1474-4422(22)00431-8. PMID: 36706773

[14] Daood R Naffaa ME Azrilin O Hassan F Jeries H Chaim P . Overlapping autoimmunity and demyelination syndromes associated with TNF inhibitor therapy. Clin Rheumatol. (2026) 45:2413–20. doi: 10.1007/s10067-026-07976-5. PMID: 41652144

[15] Friedman-Korn T Rechtman A Zveik O Vaknin-Dembinsky A . Repeated MRI-negative demyelinating attacks linked to MOG-IgG antibodies and silent lesions - case series and literature review. Clin Neurol Neurosurg. (2025) 257:109111. doi: 10.1016/j.clineuro.2025.109111. PMID: 40834627

